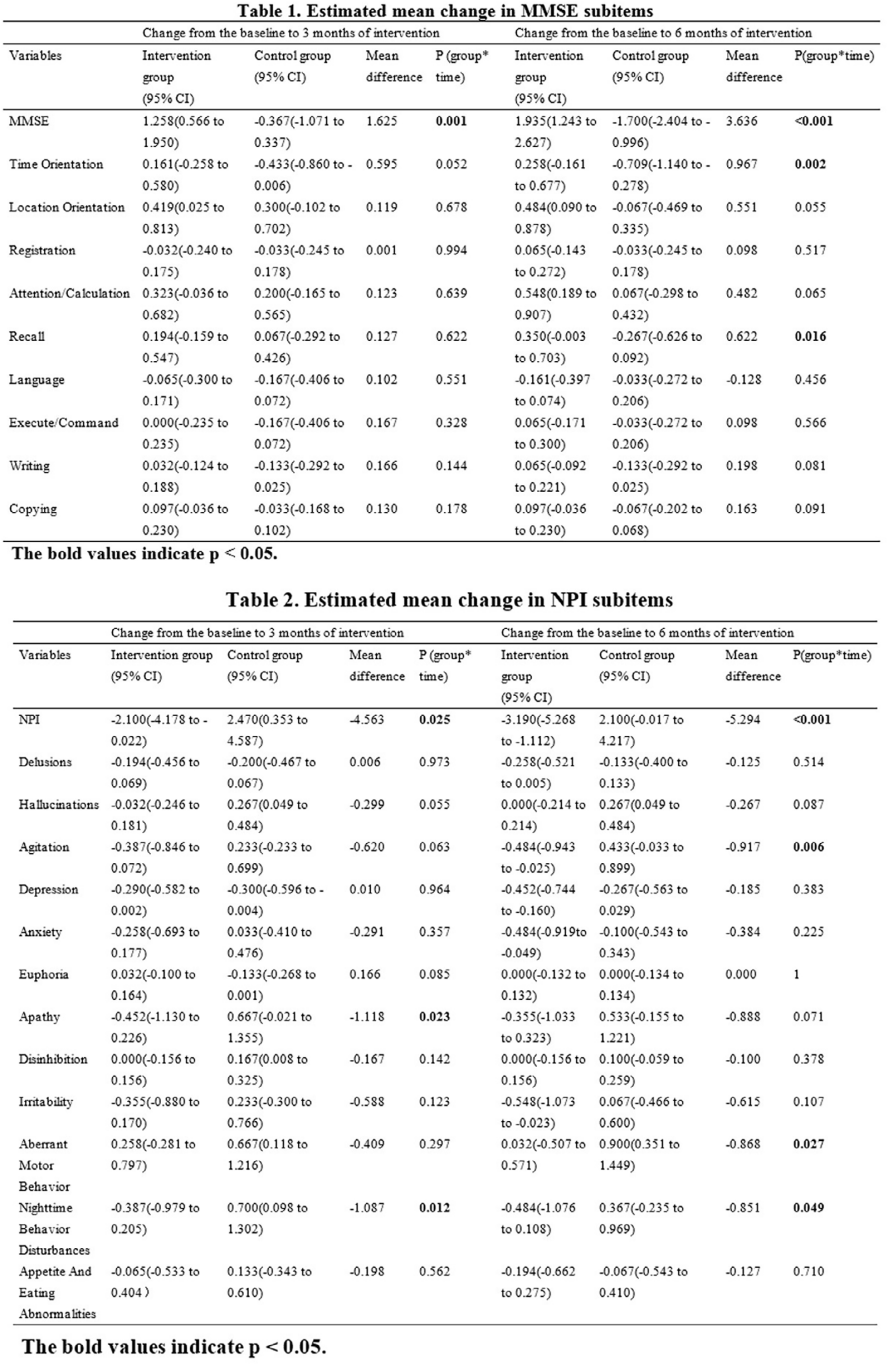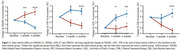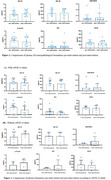# Efficacy and Safety of Music Therapy for Early Alzheimer's Disease: a Prospective, Randomized Study

**DOI:** 10.1002/alz70858_102016

**Published:** 2025-12-24

**Authors:** Shuai Liu, Junyi Wang, Yong Ji

**Affiliations:** ^1^ Department of Neurology, Tianjin Huanhu Hospital, Tianjin Key Laboratory of Cerebrovascular and neurodegenerative diseases, Tianjin dementia institute, Tianjin, Tianjin, China; ^2^ Clinical College of Neurology, Neurosurgery and Neurorehabilitation, Tianjin Medical University, Tianjin, Tianjin, China

## Abstract

**Background:**

Music therapy, as a non‐pharmacological intervention, have received attention in Alzheimer's disease (AD). While its exact mechanism in relation to AD is still not fully understand, there are few available evidence that shed light on the efficacy of biomarkers. In present study, we explored the impact of music therapy on cognitive function, behavioral and psychological symptoms of dementia, activities of daily living, the care burden of caregiver with early AD, as well as the mechanism exploration of plasma biomarkers.

**Method:**

In this randomized controlled trial, 61 people with definitive diagnosis of mild cognitive impairment due to AD and mild AD were recruited using the PET scanning to observe the deposition of beta‐amyloid proteins in the brain. Neuropsychological assessments (MMSE, ADL, NPI, CDR, ZBI) and plasma biomarkers (Aβ1‐42, Aβ1‐40, Aβ 1‐42/40 ratio, pTau181, GFAP and NfL) were assessed at baseline and post‐intervention. Primary outcome was change in cognition and neuropsychology, which were examined using generalized linear mixed models.

**Result:**

After 3 months intervention, the intervention group showed significant group*time interaction in MMSE, NPI, ADL scores relative to the control group (*p* = 0.001, *p* = 0.025, *p* = 0.014, respectively) and remained significant after 6 months intervention. For ZBI score, statistical significance did not appear until 6 months intervention (*p* ≤0.001). In subitems, significant difference were observed of the time orientation and recall of MMSE and the agitation, apathy, aberrant motor behavior and nighttime behavior disturbances of NPI. Additionally, there are no significant differences of plasma biomarker between pre‐intervention and post‐intervention, but a trend can be found. Stratifying with analysis based on the presence of apolipoprotein ε4 allele, the data trend indicated a decrease of ptau181 level in the APOE ε4 carrier group, which showed an opposite trend with APOE ε4 non‐carrier group.

**Conclusion:**

This study suggests that music therapy is effective in enhancing neuropsychological symptoms and reducing care burden. Although the difference was not statistically significant in plasma biomarker, there was a trend of change. Music therapy can be recommended as a complementary treatment to early AD.